# Roles of Reactive Oxygen Species in the Spermatogenesis Regulation

**DOI:** 10.3389/fendo.2014.00056

**Published:** 2014-04-22

**Authors:** Giulia Guerriero, Samantha Trocchia, Fagr K. Abdel-Gawad, Gaetano Ciarcia

**Affiliations:** ^1^Department of Biology, Università degli Studi di Napoli Federico II, Napoli, Italy; ^2^CIRAM, Università degli Studi di Napoli Federico II, Naples, Italy; ^3^Department of Water Pollution Research, Centre of Excellence for Advanced Science, National Research Center (NRC), Giza, Egypt

**Keywords:** spermatogenesis, reactive oxygen species, antioxidants, selenium, healthy reproduction

## Abstract

Spermatogenesis is a complex process of male germ cells proliferation and maturation from diploid spermatogonia, through meiosis, to mature haploid spermatozoa. The process involves dynamic interactions between the developing germ cells and their supporting Sertoli cells. The gonadal tissue, with abundance of highly unsaturated fatty acids, high rates of cell division, and variety of testis enzymes results very vulnerable to the overexpression of reactive oxygen species (ROS). In order to address this risk, testis has developed a sophisticated array of antioxidant systems comprising both enzymes and free radical scavengers. This chapter sets out the major pathways of testis generation, the metabolism of ROS, and highlights the transcriptional regulation by steroid receptors of antioxidant stress enzymes and their functional implications. It also deals with of the advantages of the system biology for an antioxidant under steroid control, the major selenoprotein expressed by germ cells in the testis, the phospholipid hydroperoxide glutathione peroxidase (PHGPx/GPx4) having multiple functions and representing the pivotal link between selenium, sperm quality, and species preservation.

## Introduction

Spermatogenesis appears to be a fairly conserved process throughout the vertebrate series. The balance between spermatogonial stem cell self-renewal and differentiation in the adult testis grants cyclic waves of spermatogenesis and potential fertility. These replicative processes imply a highest rate of mitochondrial oxygen consumption and reactive oxygen species (ROS) generation. Enzyme complexes of the respiratory chain of the oxidative phosphorylation, localized on the crests of the mitochondria, as the xanthines, the nicotinamide adenine dinucleotide phosphate (NADPH) oxidase and cytochrome P450, represent a source for a variety of ROS. As known, ROS are free radicals and/or oxygen derivatives that include superoxide anion, hydrogen peroxide, hydroxyl radical, lipid hydroperoxides, peroxyl radicals, and peroxynitrite. They have a dual role in biological systems, both beneficial than harmful depending on their nature and concentration as well as location and length of exposure (1). In this mini-review, we focused our attention on the relevance of ROS role in the spermatogenesis.

## Reactive Oxygen Species and Testis Mechanistic Antioxidative and Redox Defense

**Figure 1:**
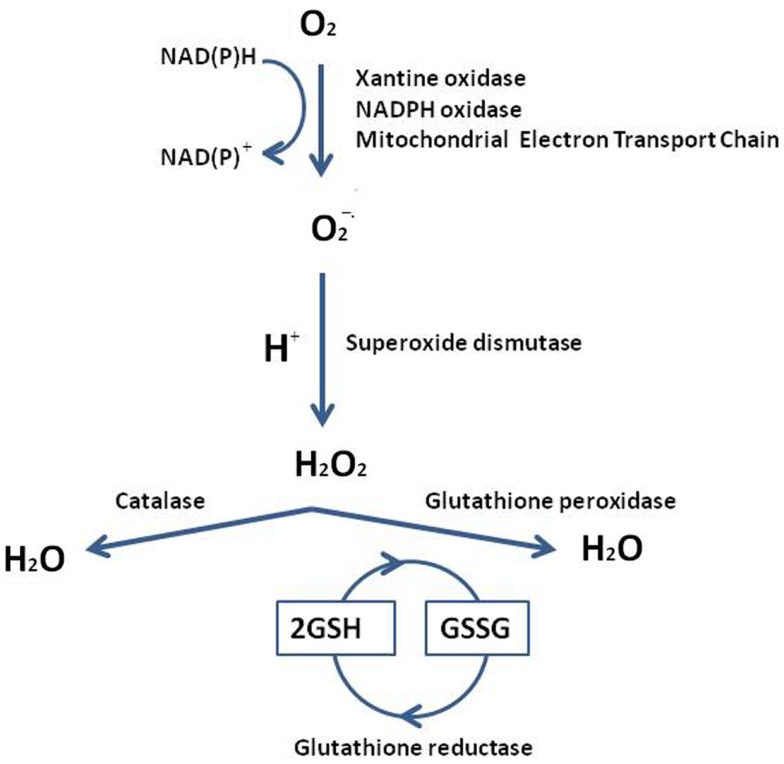
**Reactive oxygen species generation and the mechanistic antioxidative and redox defense**. The testis overexpression of ROS accelerates a response by the superoxide dismutase (SOD), glutathione peroxidase (GPx), and the glutathione-*S*-transferase (GST). The resulting oxidation product is recycled by glutathione reductase (GR), which transforms the oxidized glutathione (GSSG) back to reduced form of glutathione (GSH) [from Ref. (13)].

Reactive oxygen species are involved in all cell physiological processes. In testis, they may be beneficial or even indispensable in the complex process of male germ cells’ proliferation and maturation, from diploid spermatogonia through meiosis to mature haploid spermatozoa (2). Conversely high doses, and/or inadequate removal of ROS caused by several mechanisms, i.e., ionizing radiation, bioactivation of xenobiotics, inflammatory processes, increased cellular metabolism, activation of oxidases, and oxygenases, can be very dangerous, modifying susceptible molecules including DNA, lipids, and proteins. In addition, testis as tissue, containing large quantities of highly unsaturated fatty acids (particularly 20:4 and 22:6), results vulnerable to ROS attach. The low oxygen tension that characterizes this tissue may be an important component of the self-defense mechanism from free radical-mediated damage during spermatogenesis and Leydig cell steroidogenesis (3); together with an elaborate array of antioxidant enzymes and free radical scavengers ensures that spermatogenic and steroidogenic functions of Leydig cells are not impacted by the overexpression of ROS. In order to have a better understanding of ROS testis’ neutralization or limitation by the antioxidant systems, we summarize the major pathways of ROS generation and the mechanistic antioxidative defense in Figure 1. Superoxide radical can be generated by specialized enzymes, such as the xanthine or NADPH oxidases, or as a by-product of cellular metabolism, particularly the mitochondrial electron transport chain, and are converted to hydrogen peroxide by the superoxide dismutase (SOD). Hydrogen peroxide, present as superoxide radical and iron, forms a more reactive form, subsequently converted in lipid peroxide. Lipid peroxide is scavenged to H_2_O by glutathione peroxidase (GPx) or glutathione-*S*-transferase (GST) (4). The SOD defense by Cu/Zn-SOD, Fe/Mn-SOD, and extracellular SOD, is generally achieved by catalase or peroxidases, such as the GPxs, which use reduced glutathione (GSH) as electron donor. Glutathione keeps cells in a reduced state, acting as electron donor for other antioxidative enzymes too, and as a source for the formation of conjugates with some harmful endogenous and xenobiotic compounds, via GST’s catalysis. Levels of the reduced glutathione (GSH) are maintained via two ATP-consuming steps, involving c-glut-amylcysteine synthetase (cGCS) and glutathione synthetase. The other option constitutes a recycling system involving glutathione reductase (GR): it reduces the oxidized glutathione (GSSG) back to GSH in an NADPH-dependent way. In the interaction of GSH with ROS, GSH serves as an electron donor. The resulting oxidation product, GSSG, is either recycled by GR via electron transfer from NADPH or pumped out of the cells. Thus, GR indirectly participates in the protection of cells against oxidative stress (5, 6). In addition to the major ROS processing enzymes, in testis small molecular weight antioxidant substances are present, protecting against oxidative damage. These factors include ions, as zinc and a wide variety of free radical scavengers, vitamins C or E, melatonin and cytochrome C (7).

## Reactive Oxygen Species and Spermatogenesis Transcriptional Control

**Figure 2:**
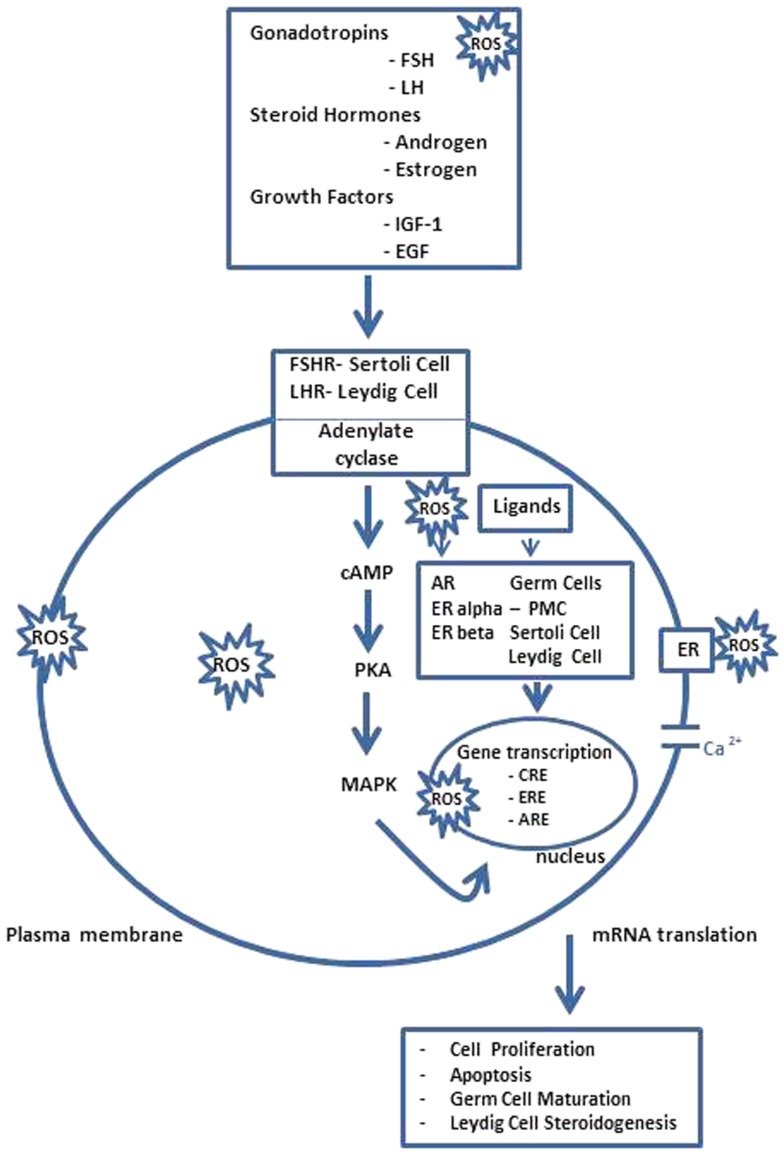
**Spermatogenesis ROS endocrine gene transcriptional regulation**. FSH acts through its receptors in Sertoli cells (FSHR) to regulate the spermatogenesis and LH stimulates androgen production by Leydig cells after binding to LHR. However, steroid hormones, i.e., androgen and estrogen, and other agents that bind or prevent binding to steroid hormone receptors, which are present in Sertoli cells, germ cells, and Leydig cells also regulate testicular function as several growth factors, e.g., insulin like growth factor-1 (IGF-1) and epidermal growth factor (EGF), acting via their receptors possibly modulate AR and ERalpha and beta-mediated pathways. The pathway, mediated by adenosine monophosphate (cAMP), appears to be the primary intracellular signaling pathway in all testicular cells and stimulates the cAMP-dependent protein kinase (PKA). Thus, testicular function is disrupted by interactions between ROS and lipids, proteins, DNA, and several signaling pathways, some acting locally, e.g., AR and ER-mediated pathways, and others indirectly by modulating hypothalamus–pituitary function. Hormonal activation of transcriptional gene activity results in changes in cell differentiation and function. PMC, peritubular myoid cell; CRE, cAMP responsive elements; ARE, androgen-responsive elements; ERE, estrogen-responsive elements [modified from Ref. (15)].

In vertebrates, the spermatogenesis is controlled by a complex network of endocrine, paracrine, and autocrine signals (8–10) Recent studies summarize different transcription factors, with a regulatory function, who modulate cellular and stage-specific gene expression. In particular, they can be subdivided in general transcription factors; nuclear receptors superfamily; transcription factors involved in testicular functions; testis-specific gene transcription, and transcriptional regulators of cell junction dynamics (11). As reported in Figure 2 in response to the hypothalamic gonadotropin hormone releasing (GnRH), the pituitary gland secretes two hormones, the luteinizing hormone (LH), and the follicle stimulating hormone (FSH), involved in the regulation of spermatogenesis, together with other important transcription factors (3). LH regulates the testosterone secretion by somatic Leydig cells located in the interstitium, between seminiferous tubules; FSH acts in Sertoli cells by stimulating signaling, gene expression, and the secretion of peptides and other signaling molecules (12) In Sertoli cells, i.e., the cAMP response element binding protein (CREB) transcription factor, an important transducer of FSH signals. Transcription factors belonging to the CREB family are involved in the regulation of gene expression in response to a number of signaling pathways inducted by ROS overexpression (13). In rat testis, alternatively, the spliced variant CREB mRNAs are spermatogenic, cycle dependent, and expressed during development of the germ and Sertoli cells, indicating that the CREB isoforms may be the major players during spermatogenesis. The transcription factor cAMP response element modulator (CREM) is highly expressed in male germ cells and regulates the expression of several post-meiotic genes, such as the transition proteins and protamines, and it likely is the key regulator of gene expression during spermatogenesis. Targeted disruption of the CREM gene blocks the differentiation program in the first step of spermiogenesis. These findings indicate a crucial role of CREM in post-meiotic germ cells differentiation, linking the action of hormonal stimuli to direct regulation of spermatogenesis genes (14). Now, it is also clear that, not only testicular somatic cells (Leydig and Sertoli cells), but also germ cells express P450arom mRNA, which is translated in a biologically active enzyme involved in the production of estrogens. Therefore, the androgen/estrogen ratio is modified in germ cells, and if testosterone is involved in the regulation of testicular functions, estrogens are also necessary not only in the control of gonadotropins secretion but also in the modulation of the Leydig cells development and steroidogenesis, as well as in the development and/or maintenance of spermatogenesis and spermiogenesis in some mammalian species (15). However, the physiological linkage between different transcription factors and ROS overexpression showed regulation by the estrogen receptor of antioxidative stress enzymes (16), the molecular target genes of these transcription factors at different stages of the seminiferous epithelial cycle are largely unknown and this shall provide an unprecedented opportunity for further investigation in the field.

## Reactive Oxygen Species and Specie Preservation

The maintenance of a high redox potential is a prerequisite to maintain the reproductive systems in a healthy state (17). Reproductive system needs ROS for reproduction, and minimizes the risk caused by ROS using antioxidative systems, such as SOD and GPx. When ROS levels exceed the scavenging capacity of the redox system, under such situations, can repair oxidized and damaged molecules using NADPH as an original electron source. In the context of defense against ROS, selenium as the glutathione (GSH) system plays key functions (18). Selenium has long been known to be necessary for the basal function of many systems of the male reproduction, also (19) is required for the synthesis of testosterone and the formation and development of the sperm (20); its deficiency affects testicular mass with damage to sperm motility, the sperm mid piece, and the shape of the sperm (21). In testis, however, most of the selenium, incorporated into proteins as selenocysteine, is associated to the enzyme phospholipid hydroperoxide GPx, PHGPx/GPx4 (22), member of the GPx named EC 1.11.1.12. PHGPx protects liposomes and biomembranes from peroxidative degradation and exhibits GPx activity on phosphatidylcholine hydroperoxides. It is, infact, able to react with hydroperoxides of fatty acids esterified in the phospholipids (23, 24); use protein thiol groups as donor substrates, to protect germ cell, by eliminating oxidative stress and reducing the levels of oxidized molecules. In rodents’ testis, PHGPx is localized in the interstitial cells of Leydig, in the nucleus of round spermatids, at the level of the cytoplasm and in the mitochondrial capsule of spermatozoa (25). Here, it is present in three different isoforms: as a cytosolic, mitochondrial, and nuclear protein (26). Functional cis-regulatory elements are identified in the promoter region of nPHGPx (27), whose expression is mediated by the transcription factor CREM-t (28). In spermatids, it is abundantly expressed as active peroxidase and during final maturation, it is transformed into a structural protein enzymatically inactive; it surrounds the helix of mitochondria in the midpiece of the sperm. The nuclear isoform, in particular, is involved in the process of the chromatin condensation, which occurs in the final steps of spermatogenesis and requires the replacement of the majority of histones, with transition proteins and protamines, essential for the stabilization of DNA and condensation of spermatocytes. These changes in location suggest that the nPHGPx can play more than a role in spermatogenesis (29). PHGPx gene expression and activity are hormone dependent processes, and they are influenced by the levels of testosterone during spermatogenesis (30). Steroid hormones do not directly activate transcription and it has been documented that, *in vivo*, testosterone promote the expression only, as a consequence of the induction of spermatogenesis (30). The study of the mechanisms of gene transcription in testis (31), suggests a crucial role of this antioxidant in male fertility and its usefulness in the screening of a potential threat to the species’ continuity (1, 32).

## Concluding Remarks

The overall objective of our mini-review was to highlight the beneficial and detrimental role of ROS that comparatively determine and influence the cyclic waves of spermatogenesis and the species preservation.

## Conflict of Interest Statement

The authors declare that the research was conducted in the absence of any commercial or financial relationships that could be construed as a potential conflict of interest.
